# Personalized medicine: paradigm shift in ALK positive non-small cell lung cancer: a case report

**DOI:** 10.1186/s13256-023-04107-5

**Published:** 2023-09-02

**Authors:** João Vasco Barreira, José Leão Mendes, Anuraj Parmanande

**Affiliations:** 1 CUF Oncologia, Lisboa, Portugal; 2 Universidade Católica Portuguesa, Lisboa, Portugal; 3 Centro Hospitalar Universitário Lisboa Central, Lisboa, Portugal; 4 Hospital SAMS, Lisboa, Portugal

**Keywords:** Oncology, Non-small cell lung cancer (NSCLC), Target Therapy, ALK-rearranged NSCLC

## Abstract

**Background:**

Since the identification of multiple therapeutic targets, as is the case of anaplastic lymphoma kinase (ALK) translocation, the paradigm of treating patients with non-small cell lung cancer (NSCLC) has improved. In order to guarantee the possibility of longer survival outcomes with a better quality of life we must invest in the determination, in suitable time, of the consensual biomarkers and in the availability of the best treatments to our patients.

**Case presentation:**

We present a case of a caucasian male in his fifth decade of life, non-smoker, who highlights the complex journey of ALK-positive patients. This particular case, demonstrates the efficacy and tolerability of the new ALK target therapies, allowing our patients to maintain their routines without compromising the effectiveness of the therapy.

**Conclusion:**

Focusing on the reality of ALK positive patients and the impact that this therapy has on the daily lives of our patients, we can contribute to the awareness of this specific pathology.

## Background

Accounting for over 1.7 million deaths per year, lung cancer is the leading cause of cancer death worldwide [1]. Based upon microscopic characteristics of tumor cells, lung cancers are mainly divided between small cell lung cancer (SCLC; 15–20%) and non-small cell Lung cancer (NSCLC; 80–85%), with the latter further subdivided into adenocarcinoma (50%), squamous cell carcinomas (30%) and large cell carcinomas. Although 5-year Overall Survival (OS) for newly diagnosed patients is currently less than 20%, survival impact rendered by the comprehension of actionable oncogene signaling pathways has led to this being a highly heterogeneous disease [2, 3]. Gene rearrangements, namely anaplastic lymphoma kinase (ALK), are among the plethora of addictive fusion oncogene mutations which have modernized the concept of personalized medicine regarding NSCLC: found in roughly 4% of NSCLC patients, the most common rearrangement occurs following the fusion between the 5′ portion of the echinoderm microtubule-associated protein-like 4 (EML4) gene and the 3′ portion of the ALK gene [4–6]. ALK positive patients are a particular group, as they include young patients, many of which having brain metastases ab initio [7]. With the following case report, we aim to outline the enhancement of both OS and quality of life (QoL) granted by ALK-targeted therapy within the rapidly evolving treatment landscape of NSCLC.

## Case presentation

**Fig. 1:**
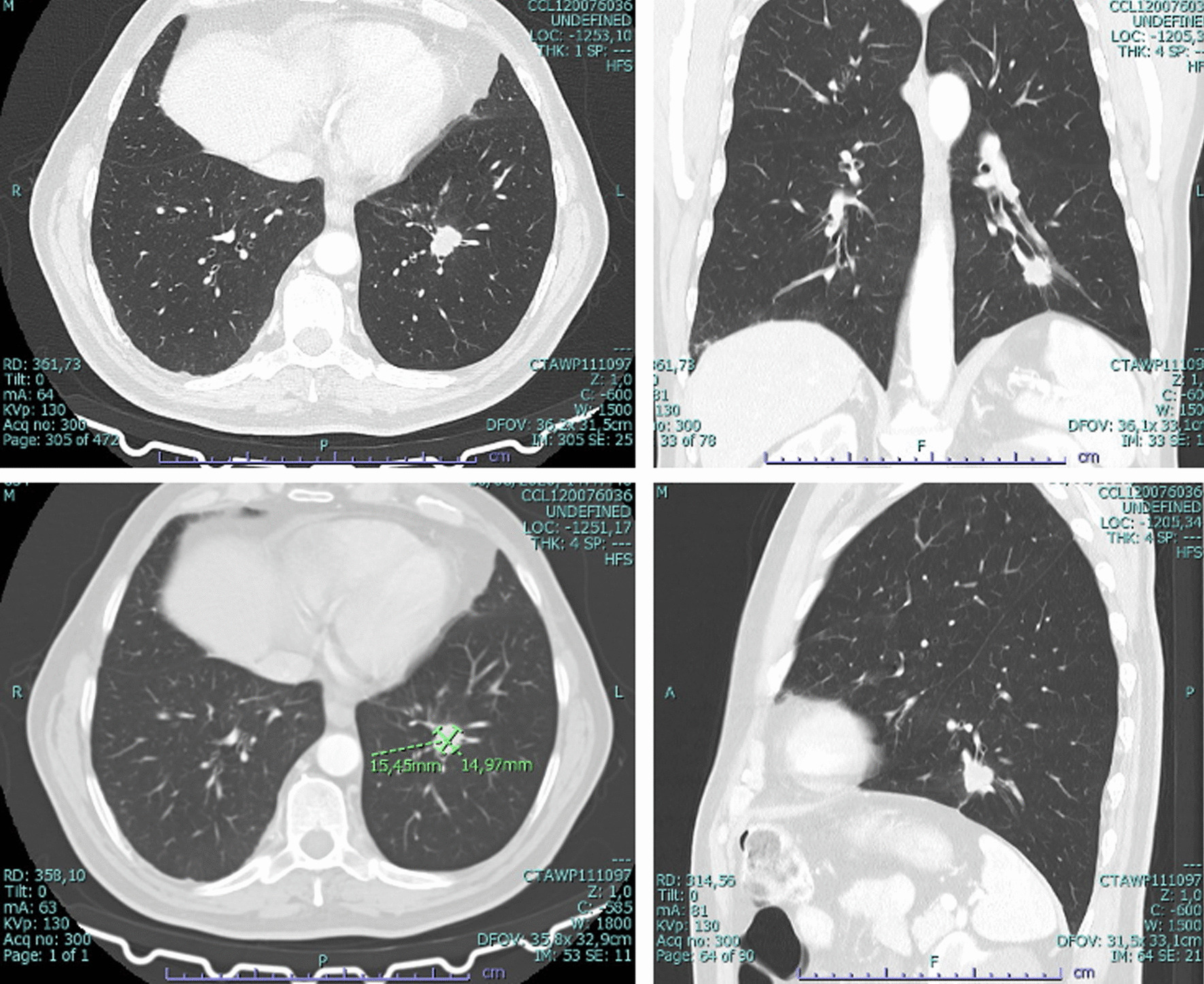
Chest Computerized tomography showing nodule in the left basal pyramid measuring about 15 × 14 mm, solid spiculated, with pleural retraction—June 2020

**Fig. 2:**
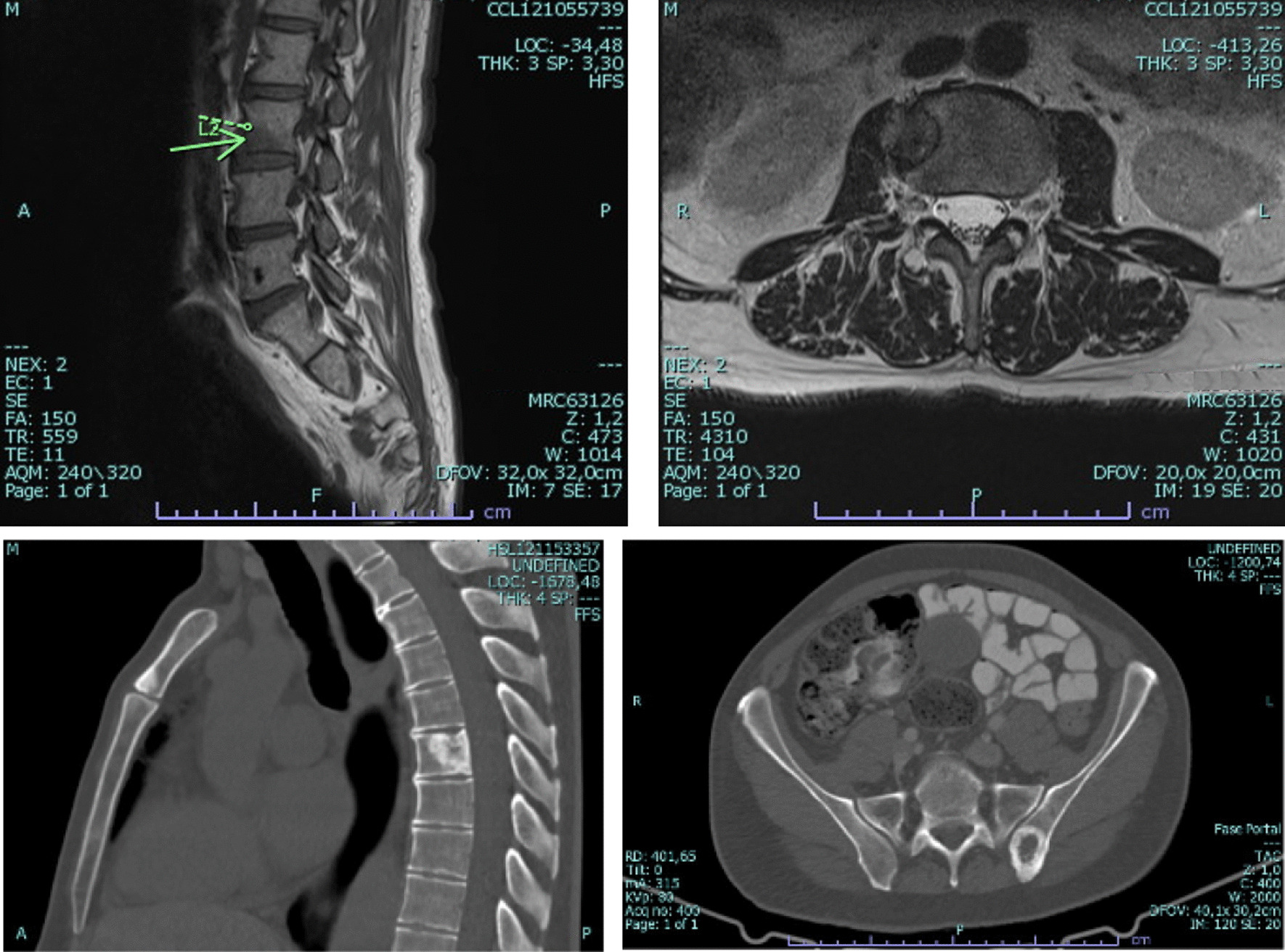
Computerized tomography and Magnetic resonance imaging of the cervical-dorsal-sacral spine revealing 4 metastatic bone lesions, again (on the arrow pointed in the image we see a de novo metastatic lesion in L2)–May 2021

A male patient in his fifth decade of life, caucasian, bank clerk, non-smoker, was admitted to our Hospital in June of 2020 following a routine CT scan, requested in a hematology consultation, showing a spiculated nodule in the left basal pyramid associated with pleural retraction (Fig. 1). He had past medical history of a myelodysplastic syndrome followed in Hematology outpatient clinic not requiring medical treatment. Cranioencephalic MRI did not show distant metastasis. Positron Emission Tomography (PET) CT scan done in August 2020 showed a left inferior lobe lesion (LIL) with SUV 5—clinical stage cT1bcN0cM0. Cytology done on LIL collected bronchoalveolar lavage was positive for poorly differentiated carcinoma, suggestive of adenocarcinoma. At this point the patient remained asymptomatic. After multidisciplinary discussion, Video-Assisted Thoracoscopic lobectomy with lymphadenectomy was performed in December 2020. Histological diagnosis of surgical specimen—pT1c pN2, G2, R0—Stage III A (AJCC 8Th Edition). After a new multidisciplinary discussion, he was proposed for adjuvant chemotherapy with Cisplatin-Etoposide—January 2021. Only two cycles were completed given that in clinical control he presented with de novo dorsal pain which triggered the performance of:

(I) **Dorsal CT** revealing two osteolytic lesions on D1 and D7, de novo (Fig. 2).
(II) **MRI Cervical-back-sacral spine**, with 4 lesions, de novo (Fig. 2).
(III) **Guided biopsy** for histological confirmation of bone metastasis.

**Histology of the left iliac wing bone**—May 2021-sample consisting of fragments of an adenocarcinoma (CK7 + , Napsin-A + , TTF1 + (focal), compatible with lung adenocarcinoma metastasis.

(IV) **Irradiation** of lesions with greater probability of complications in the short/medium term—D7, L2, left iliac, with a single fraction of 8 Gy.
(V) **Next Generation Sequencing** (NGS)—breaking of the ALK gene (2p12).
(VI) **Re-staging**—given Stage IV.
  A. Thorax **Rx**: future comparison in case of exacerbation in unscheduled consultation.
  B. Chest-abdomen-pelvis **CT** and Bone **scintigraphy**: confirms 4 small bone lesions—D1, D7, L2, left iliac.
  C. cranioencephalic **MRI**: no changes to highlight.

After multidisciplinary discussion, the patient, who had an ECOG Performance Status of 0, without target organ complaints after Radiotherapy, was started on ALK tyrosine kinase inhibitor (TKI) Alectinib 600 mg twice a day (bid) in June 2021. Two months into therapy, the patient had developed grade 3 Alanine and Aspartate Aminotransferase elevations, with no alterations seen on total bilirubin, requiring a temporary interruption and one-step TKI dose reduction at resumption (600 mg bid to 450 mg bid); second reduction to 300 mg bid was seen two months after the first one, two months after which the patient ulteriorly suspended Alectinib due to unacceptable hepatotoxicity, given that liver function tests systematically increased to a degree compatible with a grade 3, with each dose reduction performed. Following our Pharmacy and Therapeutic Commission approval, in November 2021, the patient was started on Brigatinib 90 mg once daily (id) after 4 months on Alectinib, with stable disease as best response. Given good tolerance after 1 week, Brigatinib dose was advanced to 180 mg id.

**Fig. 3:**
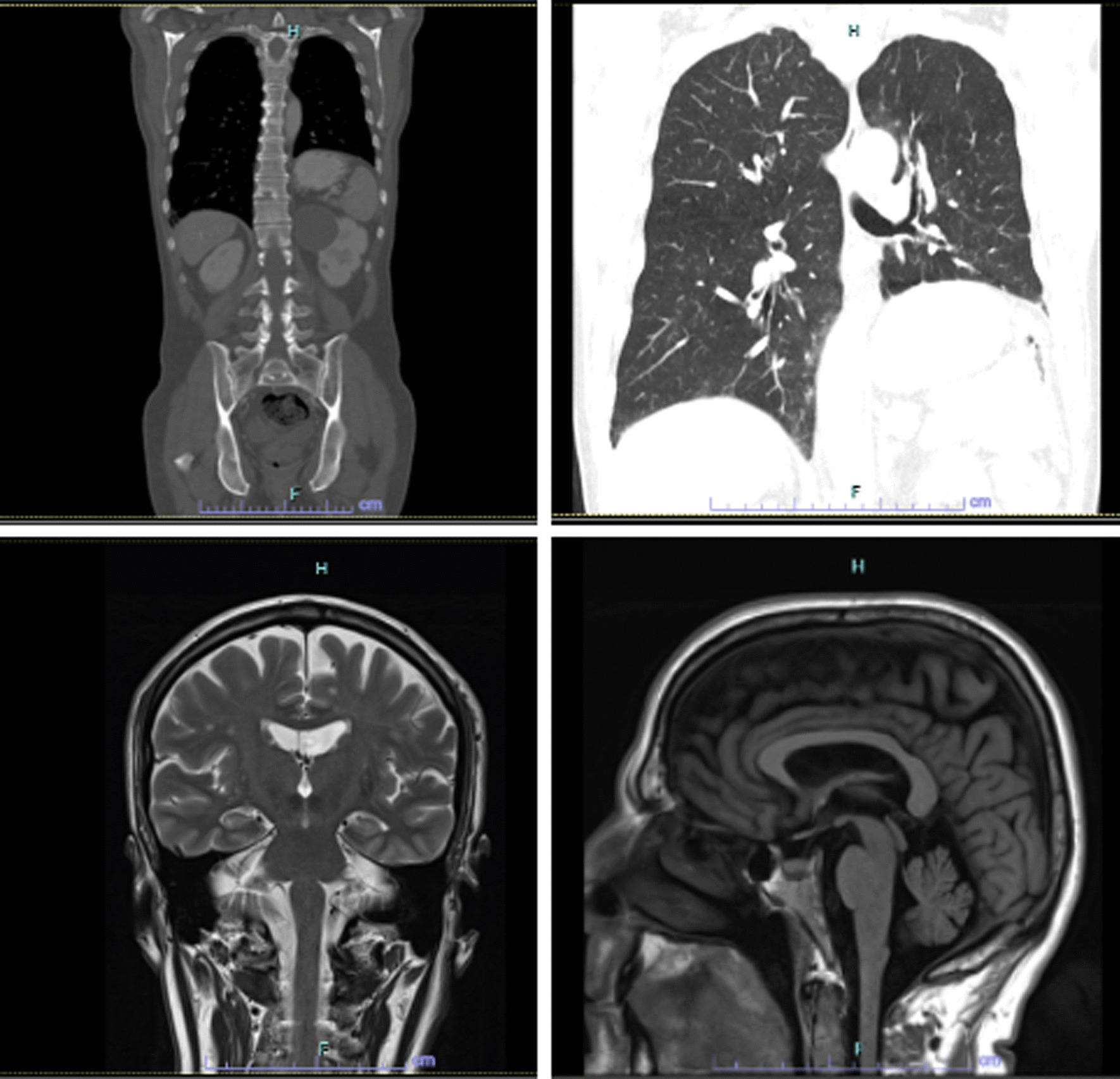
Assessment of response by imaging, Computerized tomography and Magnetic resonance imaging, demonstrating stable disease according to RECIST v1.1 criteria—January 2023

To date, our patient remains with no clinical or laboratorial remarkable findings. Having fully recovered from anorexia and asthenia, he is currently training for the Lisbon 2023 half-marathon (Fig. 3).

## Discussion and conclusions

As seen above, NSCLC can be as lethal as is challenging its treatment. ALK gene rearrangements range between 2 and 7% within NSCLC although this percentage is higher in young, non-smoking patients with lung adenocarcinomas: worth noting, the incidence of ALK mutation patients aged under 40 years old with lung adenocarcinoma are more likely to harbor this targetable genomic alteration [8].

ALK-positive lung cancer is an acquired condition, albeit there remains uncertainty regarding its exact trigger [8].

As of current standard practice, Next-generation sequencing (NGS) permits the sequencing of a broad-spectrum of oncogene mutations, ALK fusions included, allowing clinicians to act more easily on patients’ genomic data. In parallel, new therapies that target mutated genes identified through clinical NGS are gaining approval, and novel clinical trial designs in which genetic identifiers are given equal weight to histology are up-and-coming [3]. Since the identification of multiple therapeutic targets, as is the case of ALK translocation, the paradigm of treating patients with NSCLC has metamorphosed. As we reduce the tumor burden, curbing its progress, even if temporarily, we increase OS and can impact the QoL of our patients for the better. ALK inhibitors collectively and independently have shown meaningful increase in Progression Free Survival (PFS) and Overall Response Rate (ORR) without any upsurge in toxicity (grade 3/4 adverse events) rivalled to chemotherapy. First line as well as second line treatment comparison shown a comparable prominent picture of ALK inhibitors’ efficacy [9]. ALK inhibitors visibly denote a better choice of treatment for this select group of patients. Alectinib, Brigatinib, Ceritinib and Lorlatinib (second and third generation ALK inhibitors) have all shown to be favorable first choices for ALK positive NSCLC treatment following reported superiority over both chemotherapy and Crizotinib (first-generation ALK inhibitor) in terms of PFS, ORR, and intracranial efficacy [3, 9–13].

The choice of first line TKI will depend on the patient's co-morbidities and personal experience of the multidisciplinary group. Dimension of ALK positive NSCLC management is experiencing fast development with the addition of newer ALK inhibitors and the forthcoming years look brighter for this setting of patients. The fact that they are oral therapies, not implying systematic visits to the hospital, provides better QoL for often young and professionally active patients. The clinical case presented highlights the importance of Brigatinib in the treatment of ALK positive NSCLC. It is a drug with significant antitumor activity in controlling intracranial disease and has a favorable toxicity profile [11, 14].

Patients are followed up in consultations monthly with radiological, biochemical, and close clinical surveillance, maintaining an excellent general condition. However, the good results obtained in this specific case are not transversal to all patients, reflecting that it is effectively a precision medicine in which a timely diagnosis and NGS profile can make a diametrical difference in providing the best therapeutic opportunity to our patients.

## Data Availability

Not applicable.

## Abbreviations

NSCLC: Non-small cell lung cancer
CT: Computerized tomography
MRI: Magnetic resonance imaging
PET: Positron emission tomography (PET)
VATS: Video-assisted thoracoscopic surgery
Gy: Gray, unit of radiation dose
Bid: Two times a day
NGS: *Next-generation sequencing*
